# Association of *pol* Diversity with Antiretroviral Treatment Outcomes among HIV-Infected African Children

**DOI:** 10.1371/journal.pone.0081213

**Published:** 2013-11-27

**Authors:** Iris Chen, Leila Khaki, Jane C. Lindsey, Carrie Fry, Matthew M. Cousins, Robert F. Siliciano, Avy Violari, Paul Palumbo, Susan H. Eshleman

**Affiliations:** 1 Dept. of Pathology, Johns Hopkins University School of Medicine, Baltimore, Maryland, United States of America; 2 Center for Biostatistics in AIDS Research, Harvard School of Public Health, Boston, Massachusetts, United States of America; 3 Frontier Science and Technology Research Foundation, Amherst, New York, United States of America; 4 Dept. of Medicine, Johns Hopkins University School of Medicine, Baltimore, Maryland, United States of America; 5 PHRU, Chris Baragwanath Hospital, Soweto, South Africa; 6 Depts. of Pediatrics and Medicine, Geisel School of Medicine at Dartmouth, Lebanon, New Hampshire, United States of America; University of Hawaii Manoa, United States of America

## Abstract

**Background:**

In HIV-infected children, viral diversity tends to increase with age in the absence of antiretroviral treatment (ART). We measured HIV diversity in African children (ages 6–36 months) enrolled in a randomized clinical trial comparing two ART regimens (Cohort I of the P1060 trial). Children in this cohort were exposed to single dose nevirapine (sdNVP) at birth.

**Methods:**

HIV diversity was measured retrospectively using a high resolution melting (HRM) diversity assay. Samples were obtained from 139 children at the enrollment visit prior to ART initiation. Six regions of the HIV genome were analyzed: two in *gag*, one in *pol*, and three in *env*. A single numeric HRM score that reflects HIV diversity was generated for each region; composite HRM scores were also calculated (mean and median for all six regions).

**Results:**

In multivariable median regression models using backwards selection that started with demographic and clinical variables, older age was associated with higher HRM scores (higher HIV diversity) in *pol* (P = 0.005) and with higher mean (P = 0.014) and median (P<0.001) HRM scores. In multivariable models adjusted for age, pre-treatment HIV viral load, pre-treatment CD4%, and randomized treatment regimen, higher HRM scores in *pol* were associated with shorter time to virologic suppression (P = 0.016) and longer time to study endpoints (virologic failure [VF], VF/death, and VF/off study treatment; P<0.001 for all measures).

**Conclusions:**

In this cohort of sdNVP-exposed, ART-naïve African children, higher levels of HIV diversity in the HIV *pol* region prior to ART initiation were associated with better treatment outcomes.

## Introduction

Mother-to-child transmission (MTCT) of HIV can occur *in utero*, at the time of delivery, or during breastfeeding [1]. Most HIV-infected infants have relatively homogeneous viral populations that tend to diversify over time [2]–[5]. This is consistent with studies in adults that find that HIV infection is typically initiated by only one or a few HIV variants [6]. HIV diversification is driven by its large viral population size, rapid viral turnover, error-prone replication, and frequent genetic recombination [6]. Selective pressures, such as antiretroviral (ARV) drug use and the immune response, can further affect viral diversification [5], [7]–[9].

Previous studies examining the relationship between HIV diversity and disease progression have found conflicting results. In adults, high HIV diversity in the *env* region has been associated with both rapid [10], [11] and slow [12], [13] disease progression. In infants, some studies found an association between higher *env* diversity and slower disease progression [13]–[15] while others found no association between HIV diversity and disease progression [16]. Most studies of HIV diversity have used methods based on the comparison of sequences from individual HIV variants, which is time consuming and costly. This often limits the number of individuals, samples, and genomic regions that can be analyzed. The small sample volumes that are typically collected and stored in pediatric studies may be another factor limiting the analyses of HIV diversity in infants and children. For example, the studies described above included fewer than 20 children and were restricted to analysis of the *env* region [13]–[16].

We developed an assay based on high resolution melting (HRM) technology that can be used to quantify the genetic diversity of HIV populations without sequencing [17]. The level of viral diversity in each region of the HIV genome is expressed as a single numeric HRM score [17]. These scores are highly correlated with sequence-based diversity measures obtained through traditional sequencing of HIV-derived clones [17] and next generation sequencing of HIV populations [18]. In a previous study of 31 HIV-infected infants in Uganda, we found that higher HRM scores in the *gag* and *pol* regions were associated with older age and decreased 5-year survival [4]. We also found an association between higher HRM scores (in *gag*, *pol*, and *env* regions) and older age in a separate cohort of 76 Ugandan children aged 0.6–12.4 years [5]. In addition, prolonged exposure of HIV-infected children to a non-suppressive ARV drug regimen was associated with a significant reduction in the diversity of the viral population (i.e., genetic bottlenecking) [5].

In this report, we used the HRM diversity assay to evaluate the relationship between HIV diversity in *gag*, *pol*, and *env* and response to ARV treatment (ART) among 139 African children in the P1060 multi-national, randomized, clinical trial (NCT00307151) [19].

## Methods

### Ethics statement

Written informed consent was obtained from the parents or legal guardians of all children enrolled in the study. The P1060 trial was approved by the Ministries of Health and the ethics review committees at the local study sites: University of Kwazulu-Natal Biomedical Research Ethics Administration; University of the Witwatersrand Ethics Committee; Stellenbosch University; Medical Research Council of Zimbabwe; University of Zambia Research Ethics Committee; Malawi National Health Sciences Research Committee; Uganda National Council for Science and Technology HIV/AIDS Research Committee; Kilimanjaro Christian Medical College Institutional Ethics Committee; and the Medicines Control Council. The P1060 trial was also approved by the institutional review boards of the participating institutions in the United States: University of Alabama at Birmingham; Johns Hopkins University School of Medicine; University of North Carolina at Chapel Hill; and Duke University Health System [19]. The laboratory studies described in this report were approved by the institutional review board at Johns Hopkins University School of Medicine.

### Study cohort

Cohort I of the P1060 trial enrolled 164 children (ages 6–36 months) at nine sites in six African countries (four sites in South Africa and one each in Zimbabwe, Zambia, Malawi, Uganda, and Tanzania) [19]. Children in Cohort I had been exposed to single dose nevirapine (sdNVP) at the time of birth (159 received sdNVP and 5 were exposed through maternal sdNVP dosing). Maternal use of ARV drugs other than non-nucleoside reverse transcriptase inhibitors during pregnancy was permitted. All children met the World Health Organization (WHO) criteria for ART. Children were randomized to receive an initial regimen of lamivudine (3TC) and zidovudine (ZDV) with either nevirapine (NVP) or ritonavir-boosted lopinavir (LPV/r). The primary study endpoints were virologic failure or discontinuation of the NVP or LPV/r component of the ART regimen by week 24 [19].

### Laboratory methods

CD4 cell count, CD4%, and HIV viral load were measured in real time during the P1060 trial [19]. HIV genotyping was performed retrospectively using the ViroSeq HIV Genotyping System (Celera, Alameda, CA) [19]. HIV RNA or DNA remaining from genotyping was used to prepare template DNA for HRM analysis [20]. Six regions of the HIV genome were analyzed: two in *gag* (GAG1, GAG2), one in *pol* (POL), and three in *env* (ENV1, ENV2, ENV3). The HRM diversity assay was performed as previously described [20]. DNA corresponding to each region of interest was amplified by a nested polymerase chain reaction (PCR) in the presence of an intercalating duplex-dependent fluorescent dye. The DNA samples were then melted using a LightScanner instrument (Model HR 96, BioFire Diagnostics Inc., Salt Lake City, UT), and melting was monitored by the decline in fluorescence due to the release of the dye. HRM scores were calculated using an automated software tool (DivMelt, version 1.0.2) [21]; composite scores (mean and median across the six regions) were also calculated. These scores reflect the range of temperatures over which DNA melting occurred.

### Statistical methods

Fisher's exact and Wilcoxon rank-sum tests were used to compare the characteristics of participants with and without HRM results for categorical and continuous variables, respectively. Spearman correlations for continuous variables and Kruskal-Wallis tests for categorical variables were used to determine whether HRM scores were associated with characteristics at study enrollment. Median regression was used in multivariable analyses because of outliers in the HRM scores, which could over-influence results using linear regression techniques. Multivariable analysis was performed using backwards stepwise median regression starting with any variables that were significant in the univariate analysis at the P<0.10 level. The least significant variable was sequentially dropped from the model until any remaining variables were significant at the P<0.05 level.

Cox proportional hazards regression was used to assess whether HRM scores were associated with time to virologic suppression, virologic failure, virologic failure or death, and virologic failure or discontinuation of the randomized NVP or LPV/r component of the study regimen. Virologic failure was defined previously as either not achieving virologic suppression within 24 weeks or rebounding after 24 weeks [19]. Time to study endpoints was used for analyses (rather than dichotomous endpoint outcomes) since the duration of follow-up varied among the study participants (2–120 weeks); this approach increased the power for detecting differences. Models were stratified by age group (<12 months vs. ≥12 months) and then adjusted for age within each group. In addition, because pre-treatment viral load and CD4% were associated with time to study endpoints in the primary analyses of the P1060 trial [19], models were also adjusted for these factors as well as randomized study treatment arm.

We also analyzed the relationship between age and treatment adherence, which was documented in the P1060 trial as missed doses at the 2, 4, 8, 12, 16, and 24 week visits. Perfect adherence at each study visit was defined as the caregiver reporting no missed doses of study treatment in the three days prior to the visit. Wilcoxon rank sum tests were used to compare the age distribution of the participants with and without perfect adherence at each study visit.

## Results

### Study participants

**Figure 1:**
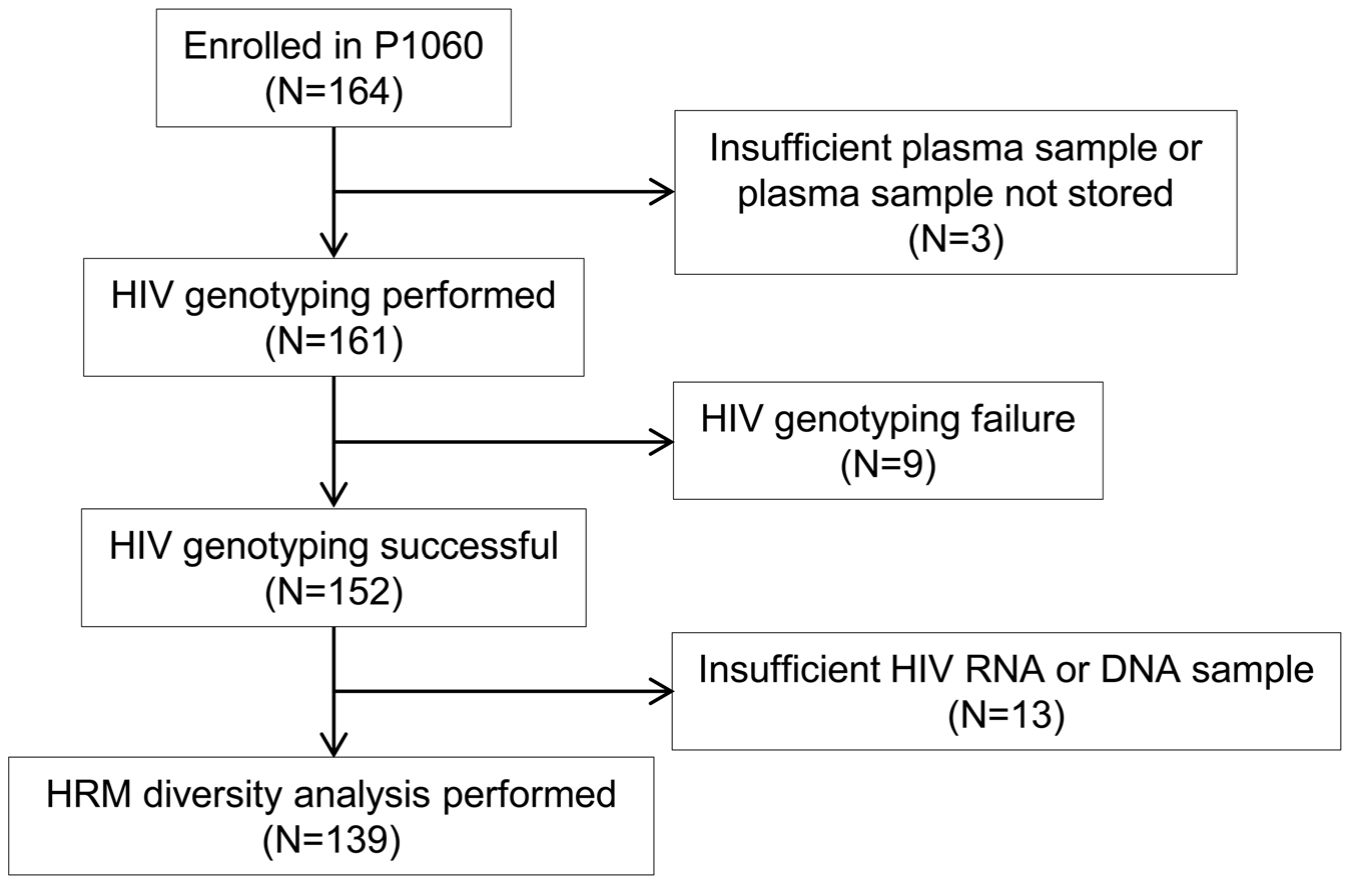
Study cohort. The high resolution melting (HRM) diversity assay was used to analyze samples from 139 (84.8%) of the 164 children enrolled in Cohort I of the P1060 trial. The 139 children had HIV genotyping results at study enrollment (prior to initiation of antiretroviral treatment) and had sufficient HIV RNA or DNA remaining after genotyping for analysis using the HRM diversity assay.

The HRM diversity assay was performed using samples collected prior to initiation of ART (pre-treatment samples). Samples were available for analysis from 139 (84.8%) of the 164 children enrolled in Cohort I of the P1060 trial (Figure 1, Table 1). The 139 children included in the study were more likely to be from South Africa (P<0.001), have no history of breastfeeding (P = 0.015), and have more advanced HIV disease as assessed by WHO stage (P = 0.047). Pre-treatment HIV viral loads were >24,400 copies/mL for all 164 children in Cohort I of P1060. No statistically significant differences were observed for CD4 cell count, CD4%, or HIV viral load among the children who were vs. were not included in the study. There were also no significant differences between the groups in the proportion of children who reached study endpoints (data not shown).

**Table 1:** Cohort I baseline characteristics overall and by inclusion in the analysis of HIV diversity.

		Included in the study			
		Total	Yes	No	
Characteristic		(N = 164)	(N = 139)	(N = 25)	P value
Country	S Africa	136 (83%)	122 (88%)	14 (56%)	**<0.001** a
	Other	28 (17%)	17 (12%)	11 (44%)	
Treatment	NVP	82 (50%)	67 (48%)	15 (60%)	0.39^a^
	LPV/r	82 (50%)	72 (52%)	10 (40%)	
Gender	Male	77 (47%)	63 (45%)	14 (56%)	0.39^a^
	Female	87 (53%)	76 (55%)	11 (44%)	
Age stratum	6 m to <12 m	123 (75%)	102 (73%)	21 (84%)	0.32^a^
	≥12 m	41 (25%)	37 (27%)	4 (16%)	
Ever breast fed	Yes	34 (21%)	24 (17%)	10 (40%)	**0.015** a
	No	130 (79%)	115 (83%)	15 (60%)	
Maternal ARV use^c^	Yes	59 (36%)	48 (35%)	11 (44%)	0.37^a^
	No	105 (64%)	91 (65%)	14 (56%)	
WHO stage	Stage I/II	72 (44%)	56 (40%)	16 (64%)	**0.047** a
	Stage III/IV	92 (56%)	83 (60%)	9 (36%)	
HIV viral load (log_10_)	Mean (sd)	5.7 (0.3)	5.7 (0.3)	5.6 (0.4)	0.43^b^
CD4 cell count	Mean (sd)	1261 (751)	1214 (693)	1522 (991)	0.20^b^
CD4%	Mean (sd)	20 (8)	20 (8)	22 (8)	0.09^b^
Any ARV resistance^d^	No	130 (86%)	119 (86%)	11 (85%)	1.00^a^
	Yes	22 (14%)	20 (14%)	2 (15%)	
	Not available	12	0	12	
HIV subtype	C	149 (97%)	134 (96%)	15 (100%)	1.00^a^
	Not C	5 (3%)	5 (4%)	0 (0%)	
	Not available	10	0	10	

The table shows baseline demographic and clinical characteristics for children who were enrolled in Cohort I of the P1060 study and were vs. were not included in the analysis of HIV diversity (see Figure 1). P values <0.05 are bolded. Abbreviations: S Africa: South Africa; NVP: nevirapine; LPV/r: lopinavir/ritonavir; m: months; WHO: World Health Organization; sd: standard deviation; HIV RNA: expressed as log_10_ copies/mL; CD4 cell count: expressed as cells/mm^3^; CD4%: CD4 cell percentage; ARV: antiretroviral.

^a^ Fisher's Exact Test.

^b^ Wilcoxon Test.

^c^ Maternal ARV drug use during pregnancy was reported for 48 infants; 47 women had used zidovudine (three had also used lamivudine [3TC]) and 1 had used 3TC and stavudine.

^d^ Twenty of the 139 infants in this study had ARV resistance at study entry; 15 had resistance to nevirapine (NVP), two had resistance to didanosine (ddI), two had resistance to NVP and ddI, and one had resistance to 3TC and emtricitabine.

### Association of HIV diversity with demographic and clinical characteristics

We evaluated the association between pre-treatment HRM scores and demographic and clinical characteristics of the study participants. Results obtained for continuous and categorical variables are shown in Tables 2 and 3, respectively. Analyses were performed using the HRM score obtained for each region of the HIV genome and for the mean and median of the six HRM scores. In univariate tests, several variables were associated with higher HRM scores (Tables 2 and 3). In multivariable median regression models using backwards selection, only age and maternal ARV drug use remained associated with any HRM scores. Older age was associated with higher POL HRM scores (P = 0.005) and also with higher mean (P = 0.014) and median (P<0.001) HRM scores. No history of maternal ARV drug use was associated with higher GAG2 HRM scores (P = 0.002).

**Table 2:** Correlation of HIV diversity and continuous demographic and clinical factors.

Variable	GAG1	GAG2	POL	ENV1	ENV2	ENV3	MEAN	MED
Age (yrs)	0.15	0.22	0.25	0.27	0.16	0.30	0.33	0.35
	(0.08)	(**0.010**)	(**0.004**)	(**0.001**)	(0.06)	(**<0.001**)	(**<0.001**)	(**<0.001**)
HIV RNA (log_10_)	0.11	−0.02	−0.13	−0.16	0.06	0.08	−0.02	−0.03
	(0.19)	(0.84)	(0.14)	(0.05)	(0.52)	(0.36)	(0.86)	(0.75)
CD4 cell count	−0.06	−0.17	−0.15	−0.07	−0.08	−0.16	−0.22	−0.23
	(0.50)	(**0.045**)	(0.09)	(0.44)	(0.38)	(0.07)	(**0.008**)	(**0.006**)
CD4%	−0.05	−0.16	−0.08	−0.08	−0.14	−0.09	−0.17	−0.23
	(0.58)	(0.06)	(0.35)	(0.33)	(0.10)	(0.32)	(**0.048**)	(**0.006**)

The table shows Spearman correlation coefficients (P values) based on ranks of continuous baseline demographic and clinical characteristics and HRM scores. CD4 and HIV-1 RNA were obtained at time of study enrollment and age was obtained at the time of randomization. HRM scores were obtained for six regions of the HIV genome (see Methods); analyses were also performed for the mean (MEAN) and median (MED) of all six HRM scores. P values <0.05 are bolded. Abbreviations: yrs: years; HIV RNA: expressed as log_10_ copies/mL; CD4 cell count: expressed as cells/mm^3^; CD4%: CD4 cell percentage.

**Table 3:** Association of HIV diversity with categorical demographic and clinical factors.

Variable	GAG1	GAG2	POL	ENV1	ENV2	ENV3	MEAN	MED
Ever breast fed	0.65	0.23	0.23	0.82	0.91	0.45	0.68	0.92
Country^a^	0.57	0.24	0.18	0.28	0.98	0.28	0.45	0.61
HIV subtype^b^	0.46	0.36	0.08	0.52	0.40	0.27	0.98	0.93
Infant ZDV use	0.18	0.33	0.56	0.07	0.34	0.08	0.09	0.06
Any maternal ARV use	0.06	**0.019**	0.15	**0.011**	0.66	0.10	**0.020**	**0.002**
Any ARV resistance	0.42	0.06	0.12	0.33	**0.033**	0.14	0.10	0.09
Gender	0.93	0.16	0.76	0.73	0.11	0.89	0.59	0.76
Randomized treatment	0.88	0.73	0.32	0.74	0.76	0.78	0.94	0.69
WHO stage^c^	0.84	0.87	0.49	0.68	0.61	0.45	0.69	0.52

The table shows the statistical significance from Kruskal-Wallis tests of the association of baseline categorical demographic and clinical characteristics and HIV diversity scores. HRM scores were obtained for six regions of the HIV genome (see Methods); analyses were also performed for the mean (MEAN) and median (MED) of all six HRM scores. P values <0.05 are bolded. Abbreviations: ZDV: zidovudine; ARV: antiretroviral; WHO: World Health Organization.

^a^ Country: South Africa vs. other countries.

^b^ Subtype C vs. other subtypes.

^c^ WHO stage I/II vs. WHO stage III/IV.

### Association of HIV diversity and clinical outcome

Ninety-four (68%) of the 139 children analyzed in this report achieved virologic suppression on ART, defined as two consecutive HIV viral load values <400 copies/mL after initiation of ART. In these analyses, statistical models were adjusted for study treatment regimen, age (years), CD4%, and HIV viral load (see Methods). Higher POL HRM scores were associated with a shorter time to virologic suppression (hazard ratio [HR]: 1.57, 95% confidence intervals [CI]: 1.09–2.26, P = 0.016, Table 4). Higher GAG1 and median composite scores were also significantly associated with shorter time to virologic suppression (Table 4).

**Table 4:** Association of HIV diversity and time to virologic suppression and study endpoints.

Region	Outcome	HR	95% CI	P value
GAG1	Virologic suppression	1.24	1.00, 1.54	**0.047**
	VF/off-treatment	0.59	0.39, 0.87	**0.009**
	VF/death	0.61	0.38, 0.99	**0.046**
	VF alone	0.62	0.37, 1.05	0.08
GAG2	Virologic suppression	1.20	1.00, 1.45	0.052
	VF/off-treatment	0.69	0.47, 1.02	0.06
	VF/death	0.75	0.50, 1.14	0.19
	VF alone	0.76	0.49, 1.19	0.24
POL	Virologic suppression	1.57	1.09, 2.26	**0.016**
	VF/off-treatment	0.35	0.19, 0.62	**<0.001**
	VF/death	0.19	0.08, 0.43	**<0.001**
	VF alone	0.16	0.06, 0.41	**<0.001**
ENV1	Virologic suppression	1.02	0.89, 1.17	0.79
	VF/off-treatment	0.88	0.59, 1.33	0.55
	VF/death	1.00	0.63, 1.59	0.99
	VF alone	1.05	0.65, 1.68	0.85
ENV2	Virologic suppression	0.98	0.85, 1.13	0.78
	VF/off-treatment	0.99	0.79, 1.25	0.95
	VF/death	1.20	0.94, 1.53	0.15
	VF alone	1.31	1.02, 1.68	**0.037**
ENV3	Virologic suppression	1.01	0.83, 1.23	0.91
	VF/off-treatment	0.86	0.65, 1.15	0.31
	VF/death	1.06	0.76, 1.48	0.72
	VF alone	1.15	0.80, 1.65	0.47
MEAN	Virologic suppression	1.24	0.92, 1.68	0.15
	VF/off-treatment	0.34	0.16, 0.72	**0.005**
	VF/death	0.57	0.24, 1.35	0.20
	VF alone	0.70	0.28, 1.76	0.46
MED	Virologic suppression	1.63	1.02, 2.62	**0.042**
	VF/off-treatment	0.31	0.15, 0.65	**0.002**
	VF/death	0.64	0.27, 1.54	0.32
	VF alone	0.76	0.29, 2.01	0.58

The table shows results from age-stratified Cox proportional hazards regression models for the association of HIV diversity with time to virologic suppression (defined as two consecutive HIV viral load values <400 copies/mL) and study endpoints, including: composite outcome of virologic failure or death (VF/death); composite outcome of virologic failure or discontinuation of study treatment (VF/off-treatment); and virologic failure alone (VF alone). Note that higher POL region diversity is positively associated with a shorter time to virologic suppression (a good clinical outcome, HR: 1.57) and is negatively associated with a longer time to study endpoints that are bad clinical outcomes (VF/off-treatment, HR: 0.35; VF alone, HR: 0.19; VF/death, HR: 0.16). In other words, higher POL region diversity is associated with improved clinical outcome for all four variables. Analyses were adjusted for study treatment regimen, age (years), CD4 cell percentage (CD4%), and HIV viral load (<750,000 vs. ≥750,000 copies/mL). HRM scores were obtained for six regions of the HIV genome (see Methods); analyses were also performed for the mean (MEAN) and median (MED) of all six HRM scores. Hazard ratios (HR) are shown as per unit increase in HRM score. P values <0.05 are bolded. Abbreviations: HR: hazard ratio; CI: confidence intervals; VF: virologic failure.

We next assessed the association of HRM scores with time to study endpoints (Table 4). Among the 139 children, 19 experienced virologic failure, one experienced virologic failure and discontinued the study ART regimen at the same study visit, five died, and 22 discontinued the study ART regimen without first experiencing virologic failure. ENV1, ENV3, and GAG2 HRM scores were not significantly associated with time to any endpoint. We found that higher POL HRM scores were associated with a longer time to all three study endpoints (P<0.001 for all three endpoints). In addition, higher GAG1 (P = 0.009), mean (P = 0.005), and median (P = 0.002) HRM scores were associated with longer time to the composite endpoint of study treatment discontinuation or virologic failure, and higher ENV2 (P = 0.037) HRM scores were associated with shorter time to virologic failure.

**Figure 2:**
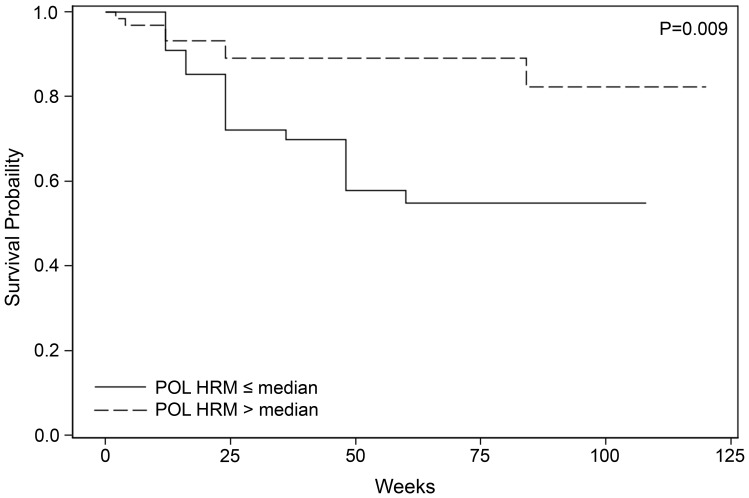
Association of POL HRM scores and time to virologic failure or death. The figure shows a Kaplan-Meier plot of time (weeks) to the composite endpoint of virologic failure or death for children with pre-treatment POL HRM scores above or at/below the median score of 4.3. The significance level is calculated from a Cox proportional hazards regression adjusted for study treatment, entry age, CD4%, and HIV viral load.

The models reported in Table 4 assume a linear relationship between HRM scores and the hazard for study endpoints. We also evaluated the association between HRM scores and time to study endpoints using a dichotomous measure (above the median HRM score vs. at or below the median HRM score for each region). Similar associations were observed for POL, mean, and median HRM scores using both continuous and dichotomous measures. Higher (above the median) POL (HR: 0.30, 95% CI: 0.15–0.61, P = 0.001, Figure 2), mean (HR: 0.44, 95% CI: 0.23–0.85, P = 0.014), and median (HR: 0.28, 95% CI: 0.13–0.57, P = 0.001) HRM scores were all associated with longer time to the composite endpoint of study treatment discontinuation or virologic failure. Higher POL HRM scores were also associated with a longer time to both virologic failure (HR: 0.20, 95% CI: 0.07–0.62, P = 0.005) and the composite endpoint of virologic failure or death (HR: 0.30, 95% CI: 0.12–0.74, P = 0.009). Higher ENV2 HRM scores also remained associated with a shorter time to virologic failure (HR: 3.75, 95% CI: 1.45–9.69, P = 0.006). In contrast, we did not observe an association between GAG1 HRM scores and any study outcome using dichotomous measures.

We considered whether the observed associations between higher *pol* diversity and better treatment outcomes may have reflected better adherence to treatment regimens among older children (who also had higher viral diversity). In the P1060 trial, treatment adherence was assessed by maternal report of missed doses at study visits 2 to 24 weeks after treatment initiation. Of the 139 children included in this study, over 92% did not have any report of missed doses. The percentage of participants with no missed doses ranged from 93.0% at week 8 (n = 114) to 98.4% at week 4 (n = 128). Furthermore, there was no indication that adherence was better among older children.

## Discussion

In this study, we identified a novel factor associated with clinical outcome on ART: a higher level of HIV diversity in the *pol* region prior to ART initiation. This association remained statistically significant in multivariate models adjusted for age, pre-treatment HIV viral load, pre-treatment CD4%, and ART regimen. The association of higher *pol* diversity with improved clinical outcomes was seen with a variety of outcome measures including: shorter time to virologic suppression after ART initiation, longer time to virologic failure, and longer time to the composite outcomes of virologic failure with either death or discontinuation of the study ART regimen. In this cohort, the definition of virologic failure took into account both initial viral suppression on ART and viral rebound (see Methods). The association between higher *pol* diversity and better clinical outcomes was observed for all study endpoints examined and when both continuous and dichotomous (above or below median group levels) diversity measures were used. Similar associations were seen for some of these comparisons when the analysis was performed using composite diversity measures (mean or median of measures from regions in HIV *gag*, *pol,* and *env*).

We also observed associations between HIV diversity measures in individual *env* or *gag* regions and study outcomes, but those findings were less consistent. For example, higher diversity in the ENV2 region was associated with shorter time to virologic failure (a worse study outcome). However, this association held for only one of the outcome measures. Higher diversity in the GAG1 region was associated with shorter time to virologic suppression and with longer time to the composite endpoint of viral failure or treatment discontinuation (better study outcomes). However, other regions of *env* (ENV1 and ENV3) and *gag* (GAG2) were not associated with any study outcome.

Results in this study are different from those of an observational cohort of Ugandan children on ART [5]. In that study, higher levels of *env* and *gag* diversity were associated with ART failure, while *pol* diversity was not. There are many differences between that study and this report that may account for the differences in our findings. This report included 139 children (vs. 76), children from four countries (vs. one country), children who were mostly infected with HIV subtype C (vs. A and D), children ages 6 to 36 months (vs. ages 6 months to 12.4 years), children who discontinued the ART regimen if they experienced viral failure (vs. continuing ART despite a lack of viral suppression), a variety of study endpoints (vs. only viral rebound), and was performed in the context of a randomized, clinical trial (vs. an observational cohort). Therefore, while the results of both studies are valid, we feel that the results from this study may be of more general relevance.

In this study of children ages 6–36 months, older age was significantly associated with higher diversity in the *pol* region. An association between higher levels of HIV diversity and older age was also observed in our previous studies of Ugandan infants and children [4], [5]. In these pediatric studies where children were known or likely to have been infected by MTCT, age serves as a surrogate for duration of HIV infection. In both children and adults, the pattern of HIV diversity and diversification has been shown to vary from person-to-person and in different regions of the HIV genome [4], [5], [20].

It is not clear why a higher level of *pol* diversity would be associated with more rapid virologic suppression or other improved clinical outcomes on ART. Because older age was also associated with higher *pol* diversity, we considered that older children might have been more adherent to their study regimens and that this may have explained the better treatment outcomes in children with higher *pol* diversity. However, there was no significant difference in treatment adherence among older vs. younger children. Therefore, it was unlikely that the association between higher *pol* diversity and better treatment outcomes was the result of better adherence among older children. We also note that the multivariate models used for the analysis were adjusted for age, in addition to other factors.

We also considered whether HIV viral load, which may be associated with viral diversity, could have confounded our results. Previous studies examining the relationship between HIV diversity and viral load found conflicting results. In adults, one study found an association between higher *env* diversity and higher viral load [22], while two other studies found an association between higher *gag* diversity and higher viral load [23], [24]. In the latter two studies, there was no association between *env* diversity and viral load [23], [24]. In this study of HIV-infected children, there was no association between viral load and diversity in *gag* or *pol* and a borderline association in *env* (p = 0.05). This is consistent with our studies of Ugandan infants where we found no association between viral load and *gag* or *pol* diversity [4], [17]. Furthermore, the association we observed between *pol* diversity and treatment outcomes in this study was independent of HIV viral load.

In theory, higher viral diversity might reflect a more vigorous immune response to HIV infection, resulting in faster or greater viral diversification and evolution. The strength of the immune response in HIV-infected infants might be influenced by the viral adaptation that appears to occur in vertical HIV transmission [25]. Viruses transmitted to infants may have already adapted to the mother's HLA alleles and immune responses. Since infants share half of the mother's HLA alleles and since most of the antibodies present in early infant infection are maternally-derived, the virus may be partially adapted to the infant's immune system at the time of transmission. In cases where the infant's virus is not as well adapted, there may be a more robust immune response, which could help contain the virus in the setting of ART. Very few studies have examined the relationship between HIV diversity and the immune response to HIV infection. One study found that HIV-infected infants with slower disease progression and higher viral diversity also had increased seroreactivity against the V3 loop of HIV [14]. We previously used the BED capture immunoassay and an avidity assay to assess antibody maturation in adults with HIV infection [26], [27]. The BED assay measures the proportion of IgG that is HIV-specific [28], while the avidity assay measures how well anti-HIV antibodies bind target antigens [29]. Further studies could use these assays to evaluate the relationship between the immune response, HIV diversity, and treatment outcome in the P1060 cohort. It should be noted, however, that the association between HIV diversity and treatment outcome in this study was most striking for the *pol* region. While antibodies that target products of the HIV *pol* gene have been described [30]–[32], those proteins are not primary targets of the humoral immune response to HIV infection.

Alternatively, higher levels of *pol* diversity prior to ART initiation may reflect properties of the viral strain, such as faster replication. The presence of ARV drug resistance mutations is often associated with reduced viral fitness [33], [34]. In theory, since high viral replication rates are one factor driving viral diversification, the diversity of viral populations might be reduced if those populations included resistant strains with reduced replication capacity. In this study, we did not see an association between pre-treatment ARV drug resistance and HIV diversity in multivariate models. Those results were based on resistance testing using a population-sequencing method that may not have detected resistant variants present at low levels [35]. We did consider the possibility that children who were previously exposed to NVP for prevention of MTCT (PMTCT) may have developed NVP resistance prior to enrollment in the P1060 trial, and that this may have led to a reduction in viral diversity, persistence of low-level NVP-resistant variants [36], [37], and worse outcomes on ART. We did not have sufficient statistical power to investigate possible interactions between treatment regimen and diversity. However, lower *pol* diversity was still associated with worse outcomes after controlling for treatment arm and other measures of HIV disease. Therefore, prior exposure to NVP for PMTCT was not likely to be responsible for the lower *pol* diversity observed in children with worse treatment outcomes.

Even though we did not see an association between ARV drug resistance and HIV diversity, differences in viral replication capacity could still explain why higher *pol* diversity was associated with better treatment outcomes. Higher replication capacity is associated with more rapid viral turnover, which could result in faster viral suppression on ART [38], as well as more rapid HIV diversification. Future studies could investigate whether differences in viral replication capacity could explain the association of higher HIV *pol* diversity with better treatment outcomes in this cohort.

Another finding in this study was the association of increased GAG2 diversity among infants whose mothers did not have a history of ARV drug use (other than sdNVP for PMTCT). Maternally-administered ARV drugs are known to be transferred to infants *in utero* and during breastfeeding [39]–[41] and can induce ARV drug resistance in infants [42], [43]. In settings where women are receiving ARV drugs while breastfeeding (e.g., ART for their own infection or a triple drug regimen for prevention of post-natal HIV transmission), this might result in a decrease in the diversity of the infant's virus (i.e., genetic bottlenecking), similar to that observed when children are maintained on non-suppressive ART [5]. These mechanisms are not likely to be relevant in this study, since most women in this cohort of P1060 did not breastfeed prior to study enrollment (83%) or during study follow-up [19].

Previous studies on HIV diversity in children have relied on sequence-based methods for diversity analysis. Those methods are more costly and labor intensive than the HRM diversity assay and also produce more complex data sets that may be more difficult to analyze than single numeric HRM scores. In contrast, the HRM diversity assay is relatively simple to perform and is well-suited for analysis of multiple genomic regions. This makes it feasible to perform larger studies such as this one, which evaluated six genomic regions in 139 children (>800 measures). The major finding in this study was that higher levels of *pol* region diversity were associated with better treatment outcomes among African children on ART. Understanding the relationship between HIV diversity and the response to ART in HIV-infected children and adults may provide new insights into factors that impact clinical responses to ART.
