# Correction: Association of *pol* Diversity with Antiretroviral Treatment Outcomes among HIV-Infected African Children

**DOI:** 10.1371/annotation/6937ccda-2f54-430d-94d0-b71d1ecc5273

**Published:** 2013-12-11

**Authors:** Iris Chen, Leila Khaki, Jane C. Lindsey, Carrie Fry, Matthew M. Cousins, Robert F. Siliciano, Avy Violari, Paul Palumbo, Susan H. Eshleman

The word "treatment" was incorrectly spelled in the Title of the article. The correct Title is: "Association of *pol* Diversity with Antiretroviral Treatment Outcomes among HIV-Infected African Children." The correct Citation is: Chen I, Khaki L, Lindsey JC, Fry C, Cousins MM, et al. (2013) Association of *pol* Diversity with Antiretroviral Treatment Outcomes among HIV-Infected African Children. PLoS ONE 8(11): e81213. doi:10.1371/journal.pone.0081213 

