# Correction: Association of *pol* Diversity with Antiretroviral Treatment Outcomes among HIV-Infected African Children

**DOI:** 10.1371/annotation/dd945f7c-c50b-461d-ab38-15e8b0966458

**Published:** 2013-12-23

**Authors:** Iris Chen, Leila Khaki, Jane C. Lindsey, Carrie Fry, Matthew M. Cousins, Robert F. Siliciano, Avy Violari, Paul Palumbo, Susan H. Eshleman

This article has been republished to correct an error in the title. 

